# Fahr's Disease Presenting with Dementia at Onset: A Case Report and Literature Review

**DOI:** 10.1155/2014/750975

**Published:** 2014-03-12

**Authors:** Rocco Salvatore Calabrò, Letteria Spadaro, Angela Marra, Placido Bramanti

**Affiliations:** IRCCS Centro Neurolesi “Bonino-Pulejo”, 98124 Messina, Italy

## Abstract

Fahr's disease (FD) is characterized by sporadic or familiar idiopathic calcification of the basal ganglia, dentate nuclei of the cerebellum, and centrum semiovale, mainly presenting with movement disorder, dementia, and behavioral abnormalities. We described a rare case of Fahr's disease presenting at onset only with behavioral and neuropsychological alterations, whose diagnosis was supposed only after a brain CT, which showed extensive bilateral calcifications in the dentate nuclei of the cerebellum and basal ganglia. Since the onset of Fahr's disease may be a dysexecutive syndrome with behavioral abnormalities, the clinical and radiological features are really important to do the appropriate diagnosis.

## 1. Introduction

Fahr's disease (FD) is characterized by sporadic or familiar idiopathic calcification of the basal ganglia, dentate nuclei of the cerebellum, and centrum semiovale [1]. People with FD frequently present with movement disorders such as rigidity, hypokinesia, tremor, choreoathetosis, and ataxia and with frontal subcortical and cortical patterns of behavioral disturbances such as psychosis, mood disorders, and dysexecutive neuropsychological syndrome [1–4]. Other neurological features are seizures or stroke-like events [2]. Although dementia is a common disorder in FD, the presentation of this disease with pure dementia (without extrapyramidal disorders) has been rarely reported [2, 5]. There is no cure for FD, nor is there a standard course of treatment. Treatment is symptomatic. While the exact pathological process is not known, it has been suggested that the hyperintense T2-weighted images seen on magnetic resonance imaging (MRI) may reflect a slowly progressive metabolic or inflammatory process in the brain, which subsequently calcifies and is probably responsible for the neurologic deficits observed. Herein we report a 71-year-old man presenting with cognitive impairment as the sole manifestation of FD.

## 2. Case Description

**Figure 1:**
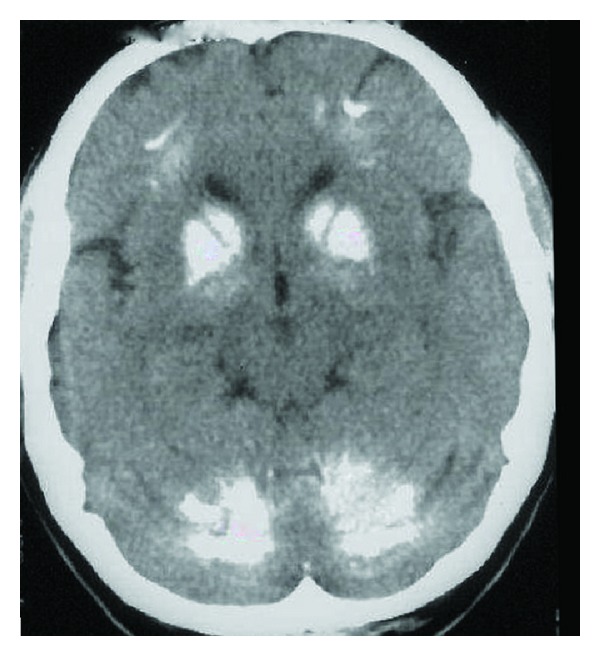
Brain TC shows calcifications of the basal ganglia and dentate nucleus of cerebellum.

A 71-year-old man came to our observation for short-term memory impairment. His family history was negative for neurodegenerative and movement disorders. He had been affected for around 15 years by diabetes mellitus and for 3 years by arterial hypertension, with good pharmacological glycemia and blood pressure control. General as well as neurological examinations were normal. At neuropsychological assessment, he presented with a dysexecutive syndrome with alterations in abstract reasoning, calculation, and sequential complex tasks, beyond mild memory impairment. Indeed, MMSE (Italian version) score was 23/30, Rey Auditory Verbal Learning test (RAVLT) was 30.30 (cut-off 28.52), Rey Auditory RAVLT Recall was 3.8 (cut-off 4.68), Attentive Matrices score was 32.75 (cut-off 30), Trial Making Test (TMT) A score was 63 (cut-off 93), TMT B score was 402 (cut-off 282), TMT B-A score was 339 (cut-off 186), and Raven's Coloured Progressive (CPM) Matrices score was 20.5 (cut-off 18). He performed daily living activities and had no behavioral disorders. Standard blood tests, including calcium and phosphorus, as well as the hormonal profile, including thyroid hormones, parathormone, and vitamin D, were normal. At one-year follow-up, the patient started losing memory daily and presenting an aggressive behavior; moreover, he had to be helped in the performance of many of the activities of daily living. Finally, in the last period, he became totally unable to perform daily living activities, with decreased short- and long-term memory and language deficits, with an important memory deficit and a severe dysexecutive syndrome. The MMSE score got as low as 11/30, RAVLT Learning was 12.30, RAVLT Recall was 0, and Attentive Matrices score was 5.75, whilst TMT and CPM were not administered. Moreover, a clear extrapyramidal syndrome was also evidenced. The computed tomography showed extensive bilateral calcifications in the dentate nuclei of the cerebellum and basal ganglia (Figure 1) and bilateral occipital silent brain infarctions.

## 3. Discussion

This is a rare case of FD presenting solely with cognitive and behavioral impairments. Indeed, to the best of our knowledge, only a few cases with the same clinical features have been described so far. The first case of FD with a pure and presenile dementia without either extrapyramidal symptoms or metabolic abnormalities has been reported by Modrego and coworkers. The authors described a patient showing bilateral striopallidodentate calcinosis and diffuse cortical atrophy predominantly in parietotemporal areas and mild periventricular hyperintensities [5]. The patient by Benke et al. presented with a neurologically “asymptomatic” FD with rapidly progressive cognitive and behavioral disorders [2]. In particular, the patient's dysexecutive syndrome was severe whereas memory and attention were only grossly/roughly compromised. Intriguingly, within twelve-month follow-up, the authors observed a rapid global deterioration, with PET findings (a reduction in glucose metabolism in the basal ganglia and frontal brain) correlating with neuropsychological symptoms.

In both case reports, as in our patient, the first cognitive symptoms concern a dysexecutive syndrome with a rapidly global cognitive deterioration without any specific neurological patterns. Moreover, the brain metabolism studied by Benke et al. [2] may explain the cognitive and behavioral problems in FD with dementia and without or with mild parkinsonian symptoms. Thus, the CT findings are really important for a correct differential diagnosis with respect to the frontotemporal dementia (FTD). Indeed, a patient by Weisman et al. [6] had a wrong diagnosis of FTD, disconfirmed only after autopsy. Finally, Lam et al. also [7] described two cases of FD with minimal extra pyramidal symptoms and specific neuropsychological deficit in frontal lobe tasks and in memory.

FD is a rare condition with specific neuroradiological features but numerous clinical manifestations. In the literature, there are 35 terms to describe bilateral calcification involving striatum, pallidum, and dentate nucleus, commonly referred to as Fahr's disease [1].

In “Fahr's Disease Registry,” the common manifestation was movement disorders (55%), in particular parkinsonism (57%), while the hyperkinetic movement disorders accounted for the rest; cognitive impairment was the second most common manifestation followed by cerebellar impairment and speech disorder. Overlap of neurologic manifestations such as movement disorder associated with cognitive impairment and cerebellar signs were often present [4]. Other minor overlapping manifestationsinclude pyramidal signs, psychiatric disorders, sensory changes, and pain [1]. In the few cases of FD with pure dementia without movement disorders, the neuropsychological evaluation disclosed a dysexecutive syndrome with behavioral problems becoming dementia, as also showed by MRI [5] and PET images [2]. It is interesting to note the use of also the diagnostic category “diffuse neurofibrillary tangles with calcification” (DNTC) as a form of progressive dementia characterized by temporal or frontotemporal atrophy with neuronal loss and astrocytosis, neurofibrillary tangles, and Fahr-type calcification, but no senile plaques in the cerebral cortex [8–14]. DNTC is a rare entity, largely confined to Japan. However, as suggested by Modrego et al. [5], the most appropriate diagnosis for the case of FD with dementia may be DNTC, although* in vivo* diagnosis is still controversial [14]. The present case describes FD with dementia without any other specific neurological signs, with regard to the extrapyramidal ones. According to the few reported cases (Table 1), the first neuropsychological signs were a dysexecutive syndrome with behavioral disorders, and, therefore, these clinical and radiological features are really important to do the most appropriate diagnosis and to differentiate FD from FTD.

## 4. Conclusions

Since individuals with FD may present at onset only with cognitive dysfunction without extrapyramidal signs, physiciansshould consider this complex syndrome when counseling patients with mental deterioration and behavioral abnormalities.

## Figures and Tables

**Table 1:** Summary of FD case with pure dementia.

Source	Country	Type of event	Age at assessment	Direct exposure group	Timing	Type of study	Movement symptoms	Misure information
Benke et al. (2004) [2]	Austria	Dementia and Fahr's syndrome	50-year-old man	1		Case report	No	NPE, CT, RMI, and FDG-PET
Modrego et al. (2005) [5]	Spain	Dementia and Fahr's syndrome	50-year-old man	1		Case report	No	NPE, CT RMI
Weisman et al. (2007) [6]	USA	Dementia and Fahr's syndrome	66-year-old man	1	10 years	Retrospective case report	5 years before onset	NPE,CT
Lam et al. (2007) [7]	Hong Kong	Fahr's syndrome; frontal lobe syndrome	38-year-old man and 59-year-old man	2	5 years	Case report	Minimal	NPE, CT
